# Supplementary material for: Erectile dysfunction and its associated factors among the male population in Adigrat Town, Tigrai Region, Ethiopia: A cross-sectional study
Source: PLoS One. 2021 Mar 19;16(3):e0242335. doi: 10.1371/journal.pone.0242335 (PMC7978351; doi:10.1371/journal.pone.0242335)
Supplement: S1 File — (DOCX) [file pone.0242335.s001.docx]

# Adigrat University

# College of Medicine and Health Science

# Department of Psychiatry

**Annex I: Information Sheet**

Dear Participants:

My name is _________________; I am hereby in the behalf of Haftom Tesfay who is an Adigrat University, college medicine and health science. This letter serves to ask consent from you to take part in this research. Different studies in different countries showed that erectile dysfunction has a significant impact on the quality of life as a result of missed opportunities for medical and psychological interventions. The purpose of this study is to assess the prevalence and associated factors of erectile dysfunction among male population. This will be a critical input for policymakers and institutions involved in care and support for persons with erectile dysfunction. Your participation in this research is voluntary. If you decide not to participate there will be no negative consequences for you. Your participation on this study is very important for the achievement of the study and for paving the way for the integration of mental health service in the care of erectile dysfunction thereby increasing the quality of care for this population. There is no any risk that will come to you because of your participation in this study. All the responses given by you and results obtained will be kept confidential using coding system whereby no one will have access to your response. You are not expected to give your name or phone number. Without permission from you and legal body, any part of this study will not be disclosed to third person. You have full right to refuse and withdrawal to participate in this study if you don't wish. The interview period will take about 30 minutes. If you are willing to participate in this study, you need to understand and sign the agreement form, and then you will be asked to give your responses to data collectors.

Name of principal investigator: Haftom Tesfay Geremedhin

Phone no: 0914272630

Are you volunteered to participate in the interview? Yes No

**ANNEX II: INFORMED CONSENT AND QUESTIONNAIRE**

**INFORMED CONSENT**

I have been briefly informed about the study and I clearly understood the objective. Since it doesn't affect my personal life, I agreed to take part in the study. Consequently, I here approve my consent to take part in the study as an interviewee with my signature.

Agreed to participate, sign and proceed to interview

Not agreed to participate, thank the respondent and end the interview

Signature ____________________ Date ______________________

**English version questionnaire**

Adigrat University College of Medicine and Health Science, Questionnaires on prevalence of sexual dysfunction and associated factors among male population in Adigrat Town.

**Section I: Questionnaires regarding Socio-demographic characteristics of the respondent**

| S.No | Questionnaires | Alternative response | Coding |
| --- | --- | --- | --- |
| 101 | How old are you? | Age in years-------------- |  |
| 102 | What is your religion? | Orthodox | 1 |
|  |  | Muslim | 2 |
|  |  | Protestant | 3 |
|  |  | Catholic | 4 |
|  |  | Others……. | 5 |
| 103 | What is your marital status? | Single | 1 |
|  |  | married | 2 |
|  |  | Divorced | 3 |
|  |  | Widowed | 4 |
|  |  | If married, how many children do you have? ….. |  |
| 104 | What is your ethnicity? | Oromo | 1 |
|  |  | Amhara | 2 |
|  |  | Somali | 3 |
|  |  | Harari | 4 |
|  |  | Others ---------- | 5 |
| 105 | What is your educational level? | Can’t write and read | 1 |
|  |  | 1-4 grade | 2 |
|  |  | 5-8 grade | 3 |
|  |  | 9-10 grade | 4 |
|  |  | College and above | 5 |
| 106 | What is your job? | Farmer | 1 |
|  |  | Merchant | 2 |
|  |  | Government employee | 3 |
|  |  | Specify other_______ | 5 |
| 107 | How much on average do you think your Monthly Income?(ETB) |  |  |

**Section II: Questions to assess social support (SSQ)**

The following 3 questions ask about how you experience your social relationships. The inquiry is about your immediate personal experience.

| S.no | Social Support Questionnaire | Response |
| --- | --- | --- |
| SSQ.201 | How many people are so close to you that you can Count on them if you have serious personal problems (choose one option)? | 1.None  2.1 or 2  3.3-5  4.More than 5 |
| SSQ.202 | How much concern do people show in what you are doing (choose one option)? | 1. No concern and interest  2.Little concern and interest  3. Uncertain  4. some  5. a lot |
| SSQ.203 | How easy is it to get practical help from friends or dorm-mates’ if you should need it (choose one option)? | 5.Very easy  4. Easy  3. Possible  2. Difficult  1. Very difficult |

### Section III: Questions to assess substance use (SUQ)

| 301 | Have you ever used khat in your life? | Yes |  |  |
| --- | --- | --- | --- | --- |
|  |  | No |  |  |
| 302 | Have you used khat in the last 03 months? | Yes |  |  |
|  |  | No |  |  |
| 303 | Have you ever used alcohol drinks in your life? | Yes |  |  |
|  |  | No |  |  |
| 304 | Have you used any kind of alcohol drinks in last 03 months? | Yes |  |  |
|  |  | No |  |  |
| 305 | Have you ever used tobacco products? | Yes |  |  |
|  |  | No |  |  |
| 306 | Have you used any kind of tobacco products in the last 03 months? | Yes |  |  |
|  |  | No |  |  |

**Section IV: Question to assess physical activity**

| 401 | Have you been doing at least 150 minutes of moderate-intensity aerobic physical activity throughout the week or doing at least 75 minutes of vigorous-intensity aerobic physical activity throughout the week? | Yes |  |  |
| --- | --- | --- | --- | --- |
|  |  | No |  |  |

**Section V: Questions to assess depression**

| No. | Over the last 2 weeks, how often have you been bothered by any of the following problems? | Not at all | Several days | More than half of the days | Nearly every day |
| --- | --- | --- | --- | --- | --- |
|  |  | 0 | 1 | 2 | 3 |
| 501 | Little interest or pleasure in doing things |  |  |  |  |
| 502 | Feeling down, depressed, or hopeless |  |  |  |  |
| 503 | Trouble falling or staying asleep, or sleeping too much |  |  |  |  |
| 504 | Feeling tired or having little energy |  |  |  |  |
| 505 | Poor appetite or overeating |  |  |  |  |
| 506 | Feeling bad about yourself or that you are failure or have let yourself or your family down |  |  |  |  |
| 507 | Trouble concentrating on things, such as reading newspaper or watching television |  |  |  |  |
| 508 | Moving or speaking so slowly that others could have noticed. Or the opposite-so fidgety or restless that you have been moving a lot than usual |  |  |  |  |
| 509 | Thoughts that you would be better off dead or hurting yourself |  |  |  |  |
| 510 | If you could checked of any problem, how difficult have these problems made it for you to do your work, take care things at home, or get along with other people? | Not difficult at all=0 | Somewhat difficult=1 | Very difficult=2 | Extremely difficult=3 |
|  |  |  |  |  |  |

**Section VI: questions to assess quality of life**

The following questions ask how you feel about your quality of life, health, or other areas of your life. I will read out each question to you, along with the response options. **Please choose the answer that appears most appropriate.** If you are unsure about which response to give to a question, the first response you think of is often the best one.

Please keep in mind your standards, hopes, pleasures and concerns. We ask that you think about your life **in the last four weeks.**

|  |  | Very poor | Poor | Neither poor nor good | Good | Very good |
| --- | --- | --- | --- | --- | --- | --- |
| 601 | How would you rate your quality of life? | 1 | 2 | 3 | 4 | 5 |
|  |  | dissatisfied | Dissatisfied | Neither satisfied nor dissatisfied | Satisfied | Very satisfied |
| 602 | How satisfied are you with your health? | 1 | 2 | 3 | 4 |  |
| The following questions ask about **how much** you have experienced certain things in the last four weeks. | | | | | | |
|  |  | Not at all | A little | A moderate amount | Very much | An extreme amount |
| 603 | To what extent do you feel that physical pain prevents you from doing what you need to do? | 5 | 4 | 3 | 2 | 1 |
| 604 | How much do you need any medical treatment to function in your daily life? | 5 | 4 | 3 | 2 | 1 |
| 605 | How much do you enjoy life? | 1 | 2 | 3 | 4 | 5 |
| 606 | To what extent do you feel your life to be meaningful? | 1 | 2 | 3 | 4 | 5 |
|  |  | Not at all | A little | A moderate amount | Very much | Extremely |
| 607 | How well are you able to concentrate | 1 | 2 | 3 | 4 | 5 |
| 608 | How safe do you feel in your daily life? | 1 | 2 | 3 | 4 | 5 |
| 609 | How healthy is your physical environment? |  |  |  |  |  |
| The following questions ask about how completely you experience or were able to do certain things in the last four weeks. | | | | | | |
|  |  | Not at all | A little | Moderately | Mostly | Completely |
| 610 | Do you have enough energy for everyday life? | 1 | 2 | 3 | 4 | 5 |
| 611 | Are you able to accept your bodily appearance? | 1 | 2 | 3 | 4 | 5 |
| 612 | Have you enough money to meet your needs? | 1 | 2 | 3 | 4 | 5 |
| 613 | How available to you is the information that you need in your day-to-day life? | 1 | 2 | 3 | 4 | 5 |
| 614 | To what extent do you have the opportunity for leisure activities? | 1 | 2 | 3 | 4 | 5 |
|  |  | Very poor | Poor | Neither poor nor good | Good | Very good |
| 615 | How well are you able to get around? | 1 | 2 | 3 | 4 | 5 |
|  |  | Very dissatisfied | Dissatisfied | Neither satisfied nor dissatisfied | Satisfied | Very satisfied |
| 616 | How satisfied are you with your sleep? | 1 | 2 | 3 | 4 | 5 |
| 617 | How satisfied are you with your ability to perform your daily living activities? | 1 | 2 | 3 | 4 | 5 |
| 618 | How satisfied are you with your capacity for work? | 1 | 2 | 3 | 4 | 5 |
| 619 | How satisfied are you with yourself? | 1 | 2 | 3 | 4 | 5 |
| 620 | How satisfied are you with your personal relationships? | 1 | 2 | 3 | 4 | 5 |
| 621 | How satisfied are you with your sex life? | 1 | 2 | 3 | 4 | 5 |
| 622 | How satisfied are you with the support you get from your friends? | 1 | 2 | 3 | 4 | 5 |
| 623 | How satisfied are you with the conditions of your living place? | 1 | 2 | 3 | 4 | 5 |
| 624 | How satisfied are you with your access to health services? | 1 | 2 | 3 | 4 | 5 |
| 625 | How satisfied are you with your transport? | 1 | 2 | 3 | 4 | 5 |
| The following question refers to how often you have felt or experienced certain things in the last four weeks. Never | | | | | | |
|  |  | Never | Seldom | Quite often | Very often | Always |
| 626 | How often do you have negative feelings such as blue mood, despair, anxiety, depression? | 5 | 4 | 3 | 2 | 1 |

**Section VII: Questions to assess erectile dysfunction**

| 701 | Over the last month, how often were you able to get an erection during sexual activity? | 0. | No sexual activity |  |
| --- | --- | --- | --- | --- |
|  |  | 5. | Almost always or always |  |
|  |  | 4. | Most times (much more than half the time) |  |
|  |  | 3. | Sometimes (about half the time) |  |
|  |  | 2. | A few times (much less than half the time) |  |
|  |  | 1. | Almost never or never |  |
| 702 | Over the last month, when you had erections with sexual stimulation, how often were your erections hard enough for penetration? | 0. | No sexual activity |  |
|  |  | 5. | Almost always or always |  |
|  |  | 4. | Most times (much more than half the time) |  |
|  |  | 3. | Sometimes (about half the time) |  |
|  |  | 2. | A few times (much less than half the time) |  |
|  |  | 1. | Almost never or never |  |
| 703 | Over the last month, when you attempted intercourse, how often were you able to penetrate your partner? | 0. | No sexual activity |  |
|  |  | 5. | Almost always or always |  |
|  |  | 4. | Most times (much more than half the time) |  |
|  |  | 3. | Sometimes (about half the time) |  |
|  |  | 2. | A few times (much less than half the time) |  |
|  |  | 1. | Almost never or never |  |
| 704 | Over the last month, during sexual intercourse, how often were you able to maintain your erection after you had penetrated your partner? | 0. | 0 No sexual activity |  |
|  |  | 5. | 5 Almost always or always |  |
|  |  | 4. | 4 Most times (much more than half the time) |  |
|  |  | 3. | 3 Sometimes (about half the time) |  |
|  |  | 2. | A few times (much less than half the time) |  |
|  |  | 1. | Almost never or never |  |
| 705 | Over the last month, during sexual intercourse, how difficult was it to maintain your erection to completion of intercourse? | 0. | No sexual activity |  |
|  |  | 5. | Almost always or always |  |
|  |  | 4. | Most times (much more than half the time) |  |
|  |  | 3. | Sometimes (about half the time) |  |
|  |  | 2. | A few times (much less than half the time) |  |
|  |  | 1. | Almost never or never |  |

**Section VIII: Questions to assess physical illness**

| Have you been told any physical illness by your doctor? | 1.Yes |
| --- | --- |
|  | 2.No |
| If **yes** question **No.407**, how many of them? (in number) | _____________ |
| If **yes** question **No. 407,** which at best describe your co-morbid illness? | 1. Cardiovascular disease |
|  | 2. Respiratory disease |
|  | 3. Renal disease |
|  | 4. Diabetic mellitus |
|  | 5. Hepatic disease |
|  | 6. Hypertension |
|  | 7. Neurological disease |
|  | 8.lower urinary tract symptoms |
|  | 9. Other specify…….. |

**ዩኒቨርሲቲዓዲግራት**

**ኮሌጅ ጥዕናን ሕክምናን ሳይንስ**

**ትምህረቲ ክፍሊ ሳይካትሪ**

**ናይ ስምምዕ ውዓሊ**

ኣነ ኣቶ______________________________________ ይብሃል፡፡

ኣቶ ሃፍቶም ተስፋይ ኣብ ዓዲግራት ዩንቨርስት ብመምህርነት እናሰርሑ ዝርክቡ ኮይኖም ብልዕቲ ጠጠው ናይ ምባል(ምቋም) ችግር ከምኡውን ተተሓሓዝቲ ወይም ትዛመድቲ ነገራት ኣብ ደቂ ተባዕትዮ ኣብ ዝብል ርእሲ ዙርያ ፅንዓት እናካየዱ ይርከቡ፡፡ ቅድሚ ሕዚ ዝተሰርሑ መፅናዓትታት ከም ዘርእይዎ ደቂ ተባዕትዮ ብልዕቲ ጠጠው ናይ ምባል(ምቋም) ችግር ብዝለዓለ መጠን ከም ዝጥቅዑን እዚ ድማ አብ ስነ-ልበንኦምን ማሕበራዊ ሂወቶምን ውፅኢታዊ ንከይኾኑ እንትገብር ይርአ፡፡ እዚ ዝኾነሉ ምኽንያት ድማ እቲ ፀገም ካብ ዘይምፍላጥን ግቡእ ምርመራን ሕክምናን ብዘይምርካቦም እዩ፡፡ ናይዚ ፅንዓት ዓላማ ናይቲ ፀገም መጠንን ተተሓሓዝቲ ዝኾኑ ነገራት ንምግምጋም እዩ፡፡ እዚ ድማ ሽግር ዘለዎን ደቂ ተባዕትዮ ኣብ ምንክብኻብን ድጋፍ ኣብ ምሃብን ተሳትፎ ንዝገብሩ ፖሊሲ ቐረፅትን ትካላትን ከም መንደርደሪ ሓሳብ የገልግል፡፡ ኣብዚ ምርምር ዝገብርዎ ተሳትፎ ኣብ ፈቓድ ዝተመስረተ እዩ፡፡ እዚ ደብዳበ ኣብዚ ፅንዓት ንክሳተፉ ፍቓድ ንምሕታት የገልግል፡፡ ንሶም ኣብዚ ፅንዓት ንምስታፍ ፍቓደኛ ስለዝኾኑ ብጣዕሚ ነመስግን፡፡ ንምስታፍ ፍቓደኛ እንተዘይኮይኩም ኣብኦም ዝበፅሕ ዋላ ሓንቲ ኣሉታዊ ዝኾነ ነገር የለን፡፡ ዝህብዎ ዝኾነ ዓይነት ሓበሬታ/መረዳእታ ብሚስጥር እዩ ዝትሓዝ፡፡ሽሞም ወይ ከዓ ስልኪ ቑፅሮም ክህቡ ግድን ኣይኮነን፡፡ እቲ ቃለ-መሕትት እንተበዚሑ 15 ደቒቓ እንተወሰደ እዩ፡፡ንምስታፍ ፍቓደኛ እንተኾይኖም ኣብ ናይ ስምምዕነት ቅጥዒ ክፍርሙ ብትሕትና ይላቦ፡፡፡

ፅንዓት ናይ ዘካይድ ሽም ፡- መ/ር ሃፍቶም ተስፋይ ስልኪ +251914272630

ኣብዚ ቃለ-መሕትት ንምስታፍ ፍቓደኛ ድዮም?

እወ ኣይኮንኩን

ፊርማ፡___________________________ ዕለት___________________________

**ናይ ዓርሰ ፍቃድ መረጋገፂ ቅጥዒ፡-** ናይቲ ምርምር ፅንዓት ዓላማን ኣካያይዳን ብዝግባእ ተብራህሪሁልይ እዩ፡፡ ስልዚ ብዝተብራህረሀለይ መንገዲ መሰረት እዚ ምርምር ምንም ዓይነት ሓደጋ ዘይብሉ ምካኑን ንዝገብሮ ተሳትፎ ክፍሊት ከምዘይብሉን ፈሊጠ ኣብዚ ናይ ምርምር ፅንዓት ንምስታፍ ፍቃድኛ ምካነይ ብፊርማየይ የረጋግፅ፡፡

ፊርማ ---------------------------

ዕለት ---------------------------

**Tigrigna Version Questionnaire**

**ትግርኛ ቋንቋ ቃለ- መሕትት ዝሓዘ ቅጥዒ**

**አካይዳ**: እዙይ ቃለ -መሕትት ሸውዓተ ክፋሊ አለውዎ፡፡ አብዚ መፅናዕቲ ንምስታፍ ፍቓደኛ እንተኮንኩም 15 ደቒቓ ግዜኩም መስዋእቲ ጌርኩም አብ ታሕቲ ዘለው ሕቶታት ንክትመልኡለይ ብትሕትና ይላቦ፡፡

**ክፍሊ1: ማሕበራውን ኢኮኖሚያውን ስነ ህዝባውን ዝምልከቱ ሕቶታት**

| 101 | ዕድመ? | ------------------ዓመት |
| --- | --- | --- |
| 102 | ብሄር? | 1. ትግራዋይ 2.ካሊእ ------ |
| 103 | ሃይማኖት? | 1. ኦርቶዶክስ 2.ሙስሊም 3.ፕሮተስታንት 4.ካሊእ |
| 104 | ኩነታት ሓዳር? | 1. በዓል ሓዳር 3. ሓዳር ዝፈተሐ  2. ዘይተመርዓወ 4. በዓል ገዛ ብህይወት ዘየለ |
| 105 | ክንደይ ቆልዑት አለውዎም? | 1. ምንም 2. ≤5   3. 6-10 4. ≥10 |
| 106 | ናይ ትምህርት ደረጃ? | 1.ምንባብንምፅሓፍን ዘይክእል 2.1-8^ይ^ ደረጃ 3. 9-10ይ ደረጃ 4.ኮሌጅን ልዕሊኡን |
| 107 | ኩነታት ስራሕ/ዝነጥፍሉ ስራሕ? | 1.ነጋዳይ 2.አብ ገዛ ዝውዕል  3.ሰራሕተኛመንግስቲ 4.ካሊእ----------- |
| 108 | ወርሓዊ ናይ ገዛ እቶት (ናይ ኢትዮጵያ ቅርሺ) | ………… |

**ክፍሊ2፡ቃለ መሕትት ዘለዎም ማሕበራዊ ደገፍ ንምግምጋም**

| ተ.ቁ | ሕቶታት | ግብረ መልሲ |
| --- | --- | --- |
| 201 | ከቢድ ናይ ውልቀ ሽግር ኣብ ዘጋጥሞም ግዜ ብዕኦም ዝተኣማመናሎም ንዕኦም ብጣዕሚ ቀረባ ዝኮኑ ክንደይ ሰባት ክረክቡ ይክእሉ? | 1.የለን 2.1 ወይ 2  3. 3 ክሳብ 5 4. ልዕሊ 5 |
| 202 | ብዝሰርሕዎ ነገር ካልኦት ሰባት ክንደየናይ ዝኣክል ትኩረት ይገብሩሎም/ይህብዎም? | 1. ተገዳስነት ኮነ ድልየት የብሎም 2.ብጣዕሚ ውሑድ ተገዳስነት 3. አይፍለጥን 4. ውሑዳት 5. ብዙሓት |
| 203 | ኣብ ዝደለይዎ ግዜ ካብ ጎረቤቶም ወይከዓ ካብ ኣዕርክቶም ሓገዝ ንምርካብ ክንደይ ዝኣክል ቀሊል እዩ? | 5. ብጣዕሚቀሊል 4. ቀሊል 3. ዘፀግም አይኮነን 2. አፀጋሚ እዩ 1. ብጣዕሚ አፀጋሚ እዩ |

**ክፍሊ 3፡ ብዛዕባ ዕፂ ኣጠቓቕማ ዝምልከት መሕተቲ**

|  | ሕቶታት | እወ | አይፋሉን |
| --- | --- | --- | --- |
| 301 | አብ ሂወቶም ጫት ተጠቒሞም ይፈልጡ ዶ? |  |  |
| 302 | አብ ዝሓለፉ 3 አዋርሕ ጫት ተጠቒሞም ይፈልጡ ዶ? |  |  |
| 303 | አብ ሂወቶም ኣልኮሆል ተጠቒሞም ይፈልጡ ዶ? |  |  |
| 304 | አብ ዝሓለፉ 3 አዋርሕ ዝኮነ ዓይነት አልኮሆል ተጠቒሞም ኔረም ዶ? |  |  |
| 305 | አብ ሂወቶም ናይ ትምባኾ ዘርኢ ተጠቒሞም ይፈልጡ ዶ? |  |  |
| 306 | አብ ዝሓለፉ 3 አዋርሕ ዝኮነ ዓይነት ናይ ትምባኾ ዘርኢ ተጠቒሞም ኔሮም ዶ? |  |  |

**ክፍሊ 4፡ ኣካላዊ ምንቅስቂስ ዝምልከት መሕተቲ**

|  | **ሕቶ** | እወ | አይፋሉን |
| --- | --- | --- | --- |
| 401 | ኣብ ሰሙን እንተዋሓደ ን150 ደቒቓ ማእከላይ ወይ ከዓ ን75 ደቒቓ ከ ቢድ ኣካላዊ ምንቅስቃስ ይገብሩ ዶ ነይሮም? |  |  |

| **ክፍሊ 5፡ ናይ ምድባር(ሓዘን) መለክዒ ሕቶታት** | | | | | | |
| --- | --- | --- | --- | --- | --- | --- |
|  | ኣብ ዝሓለፉ ክልተ ሰሙናት ካብ እንዝርርዝሮም ሽግራት በየናይ ትሽገሩ ክምዝነበሩ ክጥይቆም እየ፡፡ መልሶም ካብዚኦም ሓደ ይምረፁ**፡፡** **0=**ምንም(0-1 መዓልቲ) **፣1=**ሓሓሊፉ ጥራሕ**/2-6 መዓልትታት/** **፣2=**ብዝሕ ንዝበለ ጊዘ**/7-11 መዓልትታት/**  **3=** ዳርጋ ሙሉእ ብሙሉእ በቢ መዓልቱ**/12-14 መዓልትታት/** | | 0 | 1 | 2 | 3 |
| 501 | ኣብ ዝሓለፉ ክልተ ሰሙናት መዓልታዊ ስረሖም ንምስራሕ ዝነበሮም ምልዕዓል ወይ ድሌት ቀኒሱ ኔሩ ዶ? |  |  |  |  |  |
| 502 | ኣብ ዝሓለፉ ክልተ ሰሙናት ናይ ሓዘን፣ምድባር ወይ ተስፋ ናይ ምቁራፅ ስሚዒት ይስመዖም ኔሩ ዶ? | |  |  |  |  |
| 503 | ኣብ ዝሓለፉ ክልተ ሰሙናት ድቃስ በዚሕዎም ወይ ኣይወስድን ኢልዎም ተቸጊሮም ኔሮም ዶ? | |  |  |  |  |
| 504 | ኣብ ዝሓለፉ ክልተ ሰሙናት ናይ ድካም ወይ ዓቅሚ ምስኣን ስምዒት ይስመዖም ኔሩ ዶ? | |  |  |  |  |
| 505 | ኣብ ዝሓለፉ ክልተ ሰሙናት ናይ ምግቢ ድሌቶም(ሸውሃቶም) ካብ ልሙድ ንላዕሊ ቀኒሱ ወይ ወሲኩ ኔሩ ዶ? | |  |  |  |  |
| 506 | ኣብ ዝሓለፉ ክልተ ሰሙናት ንባዕሎም ምፅላእ፣ዋጋ የብለይን ማለት ወይ ንባዕለይን ስድራን ኣሕዚኔ ዝብለ ስምዒት ይስመዖም ኔሩ ዶ? | |  |  |  |  |
| 507 | ኣብ ዝሓለፉ ክልተ ሰሙናት ሓሳበን ንምእካብ ወይ ትኩረት ንምግባር ተቸጊሮም ኔሮም ዶ? ንኣብነት (ምስ ሰባት ክፃወቱ ከለዉ ትኩረት ሂቦም ምድማፅ) | |  |  |  |  |
| 508 | ኣብ ዝሓለፉ ክልተ ሰሙናት ንካልኦት ስባት እስካብ ዝፍለጦም ብ ምንቅስቃስ ወይ ዘረባ ብጣሚ ቀኒሶም ወይ ምርግጋዕ፣ ኣብ ሓደ ቦታ ዓሪፍካ ኮፍ ምባል፣ጠጠው ምባል ተቸጊሮም ኔሮም ዶ? | |  |  |  |  |
| 509 | ኣብ ዝሓለፉ ክልተ ሰሙናት ካብ ምነባር ሙ ማ ት ይሐሸኒ ኢሎም ሓሲቦም ወይ ንባዕሎም ብዝኮን ነግር ንሙግዳእ ሃሲቦም ኔሮም ዶ? | |  |  |  |  |
| 510 | ካብቶም ዝተዘርዘሩ ንሓዲኦም እወ ዘብል መልሲ እንተሂቦም; በዞም ሽግራት ምክንያት ስራሕ ንምስራሕ፣ ናይ ገዛ ሓላፍንቶም ንምውፃእ ወይ ም ሰባት ተስማዕሚዖም ንምንባር ክንድየናይ ተሸጊሮም ኔሮም? | ምንም ኣይተፀገምኩን | 1 | | | |
|  |  | ብመጠኑ | 2 | | | |
|  |  | ብጣዕሚ ተፀጊመ ኔረ | 3 | | | |
|  |  | ምሒር ብጠዕሚ ተፀጊመ ኔረ | 4 | | | |

**ክፍሊ 6፤ ኩነታት ኣነባብራ ዝምልከት(quality of life questioner)**

| ተ.ቁ | ሕቶታት |  |  |  |  |  |
| --- | --- | --- | --- | --- | --- | --- |
| 1. ብጣዕሚ ድኹም 2. ድኹም 3. ማእከላይ 4. ፅቡቅ 5. ብጣዕሚ ፅቡቅ | | | | | | |
| 601 | ናይ ህይወት ኣነባብራ ኩነታተን ክነደይ ዘኣክል ፅቡቅ እዩ ይብሉ? | 1 | 2 | 3 | 4 | 5 |
| 1. ብጠዕሚ ኣይዓገብኩን 2. ኣይዓገብኩን 3. ማእከላይ 4. ዓጊበ እየ 5. ብጣዕሚ ዓጊበ እየ | | | | | | |
| 602 | ብጥዕነኦም ክንደይ ዝኣክል ይዓግቡ? | 1 | 2 | 3 | 4 | 5 |
| 1. ብፉፁም 2. ንሽተይ ንሽተይ 3. ብመጠኑ 4. ብጣዕሚ 5.ሚሒር ብጣዕሚ | | | | | | |
| 603 | ኣካለዊ ሕማመን ክገብርዎ ወይ ክፍፅምዎ ዝግባእ ነገር ንከይፍፅሙ ብክነደይ ዘኣክል ኩነታት ይእግዶም? | 5 | 4 | 3 | 2 | 1 |
| 604 | ኣብ መዓልታዊ ሂዎቶም ዕለታዊ ዝኮኑ ንጥፈታት ንምፍፃም ሓገዝ ሕክምና ዘድልዮም ብክነደይ ዝኣክል መጠን እዩ? | 5 | 4 | 3 | 2 | 1 |
| 605 | ኣብ ህይዎቶም ክንደይ ዝኣክል ሕጉስ እዮም? | 1 | 2 | 3 | 4 | 5 |
| 606 | ንህይዎቶም ክንደይ ዝኣክል ትርጉም ይህበዎ? | 1 | 2 | 3 | 4 | 5 |
| 607 | ሓሳቦም ንምስብሳብ ክንደይ ዝኣክል ይክእሉ? | 1 | 2 | 3 | 4 | 5 |
| 608 | በብዕለቱ ክነድይ ዝኣክል ንህይንህይዎቶም ውሕስና ይስመዖም? | 1 | 2 | 3 | 4 | 5 |
| 609 | ዝነብርሉ ካባቢ ክንደይ ዝክል ጡዑይ እዩ? | 1 | 2 | 3 | 4 | 5 |
| 610 | ንዕለታዊ ምንቅስቃስ እኹል ጉልበት ኣለዎም ዶ? | 1 | 2 | 3 | 4 | 5 |
| 611 | ኣካለዊ ምስሎም ይቅበልዎ ዶ? | 1 | 2 | 3 | 4 | 5 |
| 612 | ድሌቶም ከማልእ ዝክል እኹል ቅርሺ ኣለዎም ደ? | 1 | 2 | 3 | 4 | 5 |
| 613 | ንዕለታዊ ህይወቶም ዘደልዮም ሓበሬታ ብቀሊሉ ክርክቡ ይክሉ ደ? | 1 | 2 | 3 | 4 | 5 |
| 614 | ክነድይ ዝኣክል ናይ ምዝንናይ ዕድል ኣለዎም? | 1 | 2 | 3 | 4 | 5 |
| 1. ብጣዕሚ ኣይኸልን 2. ኣይኸልን 3. ማእኸላይ 4. ይኸእል 5.ብጣዕሚ ይኸእል | | | | | | |
| 615 | ክንደይ ዝኣክል ካብ ቦታ ናብ ቦታ ምንቅስቃስ ይኸእሉ? | 1 | 2 | 3 | 4 | 5 |
| 1. ብጠዕሚ ኣይዓገብኩን 2. ኣይዓገብኩን 3. ማእከላይ 4. ዓጊበ 5. ብጣዕሚ ዓጊበ | | | | | | |
| 616 | ብኩነታት ድቃሶም ክንደይ ዝኣክል ዓጊቦም? | 1 | 2 | 3 | 4 | 5 |
| 617 | ዕለታዊ ስራሕ ብናይ ምውጋን(ምስራሕ) ክእለቶም ክንደይ ዝኣክል ዓጊቦም? | 1 | 2 | 3 | 4 | 5 |
| 618 | ንስራሕ ብዘለዎም ዓቅሚ ክንደይ ዝኣክል ዓጊቦም? | 1 | 2 | 3 | 4 | 5 |
| 619 | ብባዕሎም ክንደይ ዝኣክል ዓጊቦም? | 1 | 2 | 3 | 4 | 5 |
| 620 | ምስ ካልኦት ብዘለዎም ግላዊ ርክባት ክንደይ ዝኣክል ዓጊቦም? | 1 | 2 | 3 | 4 | 5 |
| 621 | ብፆታዊ ርክብ ህይወተን ክንደይ ዝኣክል ዓጊቦም? | 1 | 2 | 3 | 4 | 5 |
| 622 | ካብ ማሓዝኦም (ኣዕርክቶም) ብዝረክብዎ ድጋፍ ክንደይ ዝኣክል ዓጊቦም? | 1 | 2 | 3 | 4 | 5 |
| 623 | ብኩነታት ምነብሪ ገዝኦም ክንደይ ዝኣክል ዓጊቦም? | 1 | 2 | 3 | 4 | 5 |
| 624 | ብተባፃሓይነት ትካላት ጥዕና ክንደይ ዝኣክል ዓጊቦም? | 1 | 2 | 3 | 4 | 5 |
| 625 | ብግልግሎት መጓዓዝያታት ክንደይ ዝኣክል ዓጊቦም? | 1 | 2 | 3 | 4 | 5 |
| 1. ብፍፁም 2. ብጣዕሚ ሓሊፉ ሓሊፉ 3. ሓሊፉ ሓሊፉ 4. ብዙሕ ጊዜ 5.ኹሉ ጊዜ | | | | | | |
| 626 | ኣሉታዊ ስምዒታት ንኣብነትምስልቻው፣ተስፋምቁራፅ፣ፍርሒ፣ድብርቲ ብክነደይ ዝኣክል ተደጋጊምዎም? | 5 | 4 | 3 | 2 | 1 |

**ክፍሊ 7፡ ብልዕቲ(መትሎ) ጠጠው ናይ ምባል(ምቋም) ችግር ዝምልከቱ ሕቶታት**

| ተ.ቁ | ሕቶታት | ምማረፂ መልስታት | ኮድ |
| --- | --- | --- | --- |
| 701 | ብልዕተኦም ናይ ምቋም ና ኣቝሞም ናይ ምፅናሕ ምትእምማኖም ከመይ ገምግምዎ? | ብጣዕሚ ትሑት | 1 |
|  |  | ትሑት | 2 |
|  |  | ማእከላይ | 3 |
|  |  | ልዑል | 4 |
|  |  | ብጣዕሚ ልዑል | 5 |
| 702 | ብስምዒት ተላዓዒሎም ብልዕቶም ክቖም ከሎ ክንደይ ዝኣክልጊዜ ጠንካራን ልርክብ ግብረ ስጋ ዝግጁ ኔሩ? | ብፍፁም | 1 |
|  |  | ውሑድ ጊዜ (ትሕቲ ፍርቂ ጊዜ) | 2 |
|  |  | ሓሓሊፉ( ዳርጋ ፍርቂ ጊዜ) | 3 |
|  |  | ምብዛሕቲኡ ጊዜ(ልዕሊ ፍርቂ ጊዜ) | 4 |
|  |  | ኩሉ ጊዜ ወይ ዳርጋ ኩሉ ጊዜ | 5 |
| 703 | ፆታዊ ርክብ ክፍፅሙ ከለዉ፤ናብ ብልዕቲ ተፃመዲት ምስ ኣተወ ንክንደይ ዝኣክል ጊዜ ብልዐቶም ቆይሙ ይፀንሕ? | ብፍፁም | 1 |
|  |  | ውሑድ ጊዜ (ትሕቲ ፍርቂ ጊዜ) | 2 |
|  |  | ሓሓሊፉ( ዳርጋ ፍርቂ ጊዜ) | 3 |
|  |  | ምብዛሕቲኡ ጊዜ(ልዕሊ ፍርቂ ጊዜ) | 4 |
|  |  | ኩሉ ጊዜ ወይ ዳርጋ ኩሉ ጊዜ | 5 |
| 704 | ክሳብ ፆታዊ ርክቦም ዝውድኡ ብለዕቶም ቘሙ ንምፅናሕ ክንደየናይ ከቢድ ኔሩ? | ሚሒር ብጠዕሚ ከቢድ | 1 |
|  |  | ብጠዕሚ ከቢድ | 2 |
|  |  | ከቢድ | 3 |
|  |  | ዳርጋ ከቢድ | 4 |
|  |  | ከቢድ ኣይነበረን | 5 |
| 705 | ፆታዊ ርክብ ክፍፅሙ ከለዉ ክንደይ ዝኣክል ጊዜ ይረክዑ ኔሮም? | ብፍፁም | 1 |
|  |  | ውሑድ ጊዜ (ትሕቲ ፍርቂ ጊዜ) | 2 |
|  |  | ሓሓሊፉ( ዳርጋ ፍርቂ ጊዜ) | 3 |
|  |  | ምብዛሕቲኡ ጊዜ(ልዕሊ ፍርቂ ጊዜ) | 4 |
|  |  | ኩሉ ጊዜ ወይ ዳርጋ ኩሉ ጊዜ | 5 |

**ክፍሊ 8፡ ስለ ካላዊ ሕማማት ዝምልክቱ ሕቶታት**

| 801 | ቅድሚ ሕዚ ካላዊ ሕማም ኣለኩም ተባሂልኩም  ነይርኩም ዶ? | 1.እወ |
| --- | --- | --- |
|  |  | 2.ኣይተባሃልኩን |
| 802 | እነድሕር ንቁ.701 መልስኹም እወ እንድሕር ኮይኑ ክንደይ  ዝኣኽሉ ሕማማት ኣለኩም ተባሂልኩም ነይርኩም?  (ብቑፅሪ) | _____________ |
| 803 | እነድሕር ንቁ.702 መልስኹም እወ እንድሕር ኮይኑ እቲ ሕማምኩም እንታይ ይብሃል? | 1. ሕማም ልቢ |
|  |  | 2. ሕማም ስርዓተ ምስትንፋስ |
|  |  | 3. ሕማም ኩላሊት |
|  |  | 4. ሕማም ሽከር |
|  |  | 5. ሕማም ፀላም ከብዲ |
|  |  | 6. ፀቕጢ ደም |
|  |  | 7. ሕማም ስነ-ምትኒ |
|  |  | 8. ምልክታት ኣብ ታሕተዋይ ናይ ሽንቲ ክፍሊ |
|  |  | 9. ካሊእ…….. |
