# Supplementary material for: Erectile dysfunction and its associated factors among the male population in Adigrat Town, Tigrai Region, Ethiopia: A cross-sectional study
Source: PLoS One. 2021 Mar 19;16(3):e0242335. doi: 10.1371/journal.pone.0242335 (PMC7978351; doi:10.1371/journal.pone.0242335)
Supplement: Attachment — Submitted filename: Response to reviewers.docx [file pone.0242335.s003.docx]

Editors

PLOS ONE

October 07, 2020

Dear Dr. Rohrmann,

Thank you for giving us the opportunity to submit the revised draft of our manuscript entitled with “Erectile dysfunction and its associated factors among the male population in Adigrat Town, Tigrai Region, Ethiopia: A cross-sectional study” to PLOS ONE. We appreciate the time and effort that you and the reviewers have dedicated to provide your valuable feedback on our manuscript. We are grateful to you and the reviewers for the insightful comments on our paper. We have tried to revise our manuscript in accordance with the suggestions and comments provided by you and the reviewers.

On the following pages, you will find our point-by-point response to the comments made by the editor and reviewers.

Sincerely,

Haftom Tesfay Gebremedhin, MSc.

Adigrat University, Ethiopia
E-mail: [haftomtesfay30@gmail.com](mailto:haftomtesfay30@gmail.com);

Tele: +251-969 16 44 12

**Point by point responses**

| **Comments** | **Responses** |
| --- | --- |
| 1. *Please ensure that your manuscript meets PLOS ONE's style requirements, including those for file naming. The PLOS ONE style templates can be found at*   [*https://journals.plos.org/plosone/s/file?id=wjVg/PLOSOne_formatting_sample_main_body.pdf*](https://journals.plos.org/plosone/s/file?id=wjVg/PLOSOne_formatting_sample_main_body.pdf) *and* [*https://journals.plos.org/plosone/s/file?id=ba62/PLOSOne_formatting_sample_title_authors_affiliations.pdf*](https://journals.plos.org/plosone/s/file?id=ba62/PLOSOne_formatting_sample_title_authors_affiliations.pdf) | Modified according to the PLOS ONE's style requirements. |
| 2. *Please include a copy of Table 4 which you refer to in your text on page 15. (Table 1 2x)* | It was a slip of pen that we wrote “Table 4” in both the reference and the title of the table and we have corrected the mistake and revised it to “Table 3” (page 15, line 257-259). |
| 3. *The prevalence of ED is rather low. This theoretically is attributed to the generally low age of study participants. Indeed this rate is in line with studies assessing the ED prevalence in younger populations. A comment should be made in the discussion section stating that the age of the study participants is rather low and thus the true ED prevalence may be underestimated.* | We agree with this comment. Accordingly, we have stated it in discussion part (page 18, line 281-283). |
| 4. *Dyslipidemia is a common risk factor for ED. Are there any data regarding the prevalence of dyslipidemia in the study participants? And if yes, was there any association with ED prevalence and severity?* | We accepted the comment and **have included it as the limitation of the study in the limitation** section of the manuscript (page 19, line 324-326). |
| 5. *Depression is commonly associated with increased risk for ED. Drugs for the treatment of depression are often accompanied by ED as a side effect. Was there any relevant association?* | Thank you for noticing this. In fact medications used to treat depression have an effect on an individual’s sexual performance, but we omitted them. We have therefore included them as a limitation of the study (page 19, line 324-326). |
| 6. *Several drugs for the treatment of cardiovascular diseases and risk factors (such as beta-blockers and diuretics) are related with increased risk for ED. If data are available, please provide any relevant information regarding the use of such drugs and potential relation with ED prevalence.* | It is true that medications used to treat cardiovascular disease are important factors for ED, and we have included them in the limitation part of the study (page 19, line 324-326). |
| 7. *The manuscript is in need of grammatical revision. Please check.* | Checked. |
| 8. *Include the confidence interval of the prevalence in the abstract.* | We agree with this comment. Accordingly, we have included the confidence interval of the prevalence in the abstract part (page3, line 53). |
| 9. *Census 2007 is too old for a rapidly growing population. Please use predicted population.* | Thank you for pointing out this and we have updated this with the latest (2017) estimated population of the Town from the report of the Central Statistical Agency of the Federal Democratic Republic of Ethiopia (page 6, line 113-115). |
| 10. *" Who had been engaged in sexual activity in a preceding month " is a defining term for the inclusion of your study participants. This is a strong term that may have excluded many people with erectile dysfunction (ED), which would have increased the prevalence if they had been included. Do not believe that your findings could show a severely underestimated prevalence.* | The instrument (The International Index of Erectile Function Questionnaire (IIEF-5)) used to determine erectile dysfunction measures the erectile function over the last month and that is why we used sexual activity in the last moth preceding the data collection as inclusion criteria. |
| 11. *While your target study participants were individuals, you targeted at a household. This looks a bit superfluous and unnecessary. Other options will have been sought.* | Thank you for the suggestion. It was hard to get a list of male individuals in the Town. Instead of selecting individuals from the sampling frame, we used systematic randomized sampling to select study unit (households) and simple randomized sampling to select study participants if we found more than one eligible study participants. In this method, sampling error might be unavoidable, thus minimizing the error that we used the design to maximize sample size. |
| 12. *Why was "bivariate regression"? That somehow looks like a tradition. This is necessary if you have a smaller sample but more variables and want to select fewer variables. This is to counteract unstable estimates when you fit more variables into a small sample. However, you have a sufficient sample size for the number of variables. This tradition is also problematic. For example, a variable may not be significant if you perform bivariate regression, but it could have an important interaction effect if you use it with other variables. Do not follow this tradition.* | We appreciate your suggestion. We will consider it for the future. |
| 13. *Minor: I think you can reduce the content of your tables (e.g. in table 2 you can have only one column and one row for each variable. The answers 'Y/N' are not necessary. And you also make the frequency and percentage in one column)* | Great point. We have modified our tables accordingly. |
| 14. *Quality of life pie chart is not necessary.* | Agreed. We removed it. |
